# Therapeutic Cancer Vaccines in Gastrointestinal Malignancies: Advances, Challenges, and Emerging Strategies

**DOI:** 10.3390/cancers18091420

**Published:** 2026-04-29

**Authors:** Kyle Taing, Keeyon Dabirian, Aditya Shreenivas

**Affiliations:** 1 Department of Internal Medicine, Huntington Hospital, Pasadena, CA 91105, USA; keeyon.dabirian@huntingtonhealth.org; 2 Department of Medical Oncology & Therapeutics Research, City of Hope, Duarte, CA 91010, USA

**Keywords:** cancer vaccines, neoantigens, tumor microenvironment, immune checkpoint inhibitors, personalized vaccines

## Abstract

Gastrointestinal (GI) malignancies—which comprise esophageal, gastric, colorectal, hepatobiliary, and pancreatic cancers—remain a leading global cause of oncologic morbidity and mortality. The prognosis for many patients (especially those diagnosed with advanced-stage disease) remains poor despite conventional therapies—namely, surgery, chemotherapy, and radiation. Immunotherapy, however, has emerged as a new strategy in oncology, and, in particular, the advent of cancer vaccines now provides an investigational approach to improving clinical outcomes in patients with GI malignancies. This review aims to provide a comprehensive overview of multiple vaccine-based strategies developed to better target GI cancers, spanning from early preclinical studies to the most recently completed clinical trials. We first introduce the main vaccine therapy classes and the immunologic rationale underlying each. We then summarize key findings from past and ongoing trials using a cancer-type-based approach, primarily focusing on vaccine safety and immunogenicity, and commenting on limitations in overall efficacy. Finally, we identify the challenges of applying mostly early-phase trials to clinical practice as well as future directions for integrating these vaccine-based approaches into personalized treatments for GI cancer patients.

## Simple Summary

Gastrointestinal tract cancers—which target the esophagus, stomach, colon, liver, biliary system, and pancreas—continue to represent a major worldwide cause of illness and death. Many patients continue to face a poor prognosis despite surgery, chemotherapy, and radiation. In this review, we examine the development of cancer vaccines, a developing form of immunotherapy designed to stimulate the body’s own immune system to recognize and attack tumor cells. Specifically, we report on the development of vaccine-based therapies for gastrointestinal cancers, including different vaccine categories as well as results from historical and recent clinical trials, while carefully noting the exploratory nature of the findings. We also discuss ongoing challenges in this field, including the ability of tumors to suppress the immune system, as well as strategies to overcome these barriers. In summarizing the current state of cancer vaccine development, this review aims to guide ongoing research in effective vaccine strategies.

## 1. Introduction

Gastrointestinal (GI) cancers represent a wide breadth of pathology that encompasses malignancies of the esophagus, stomach, colorectum, liver, biliary tract, and pancreas. Cumulatively, they constitute a substantial portion of cancer morbidity and mortality, with the World Health Organization reporting that they account for 26% of the global cancer incidence burden as well as 35% of all cancer-related deaths [1]. Despite ongoing oncologic advances in surgery, radiotherapy, chemotherapy, immunotherapy, and targeted therapies, the prognoses for many patients remain poor, especially when diagnosed at an advanced stage with possible metastasis. Furthermore, other unfavorable factors such as tumor recurrence, tumor resistance to chemotherapy, and (often intolerable) treatment-related toxicity also continue to impede long-term survival [2,3]. As such, there remains an urgent need for novel therapeutic modalities to better target the various types of GI malignancies compared to the current standard of care.

Cancer vaccines have emerged as another method of cancer targeting in recent years. For instance, multiple vaccine-based therapies have been designed to stimulate cytotoxic T-lymphocytes against defined tumor or neoantigen targets [4]. Over the past two decades, multiple vaccine categories have been developed for GI malignancy; although still predominantly investigated in early phase trials, they have shown potential as complementary, synergistic, and single-agent therapies. This review will survey both past findings on vaccine-induced immunogenicity and more recent attempts to establish the clinical efficacy of cancer vaccines, as relevant to GI malignancies, which are among the most immunologically heterogeneous tumor groups. We will first define and examine the various classes of cancer vaccines as well as the dynamic interplay between each vaccine type and multiple components of the immune system. Next, we will catalog the clinical outcomes reported to date for vaccine therapy in each major category of GI malignancy, along with ongoing trials, while emphasizing that most trials demonstrate safety and preliminary signals of efficacy rather than established clinical benefit. Finally, we will highlight both the opportunities and challenges of integrating such vaccines into the standard of care in gastrointestinal oncology. Building on prior literature, this review provides a unique perspective through establishing a framework for GI cancer vaccines that underscores immune context, tumor heterogeneity, and therapeutic use.

## 2. Vaccine Classes

**Figure 1:**
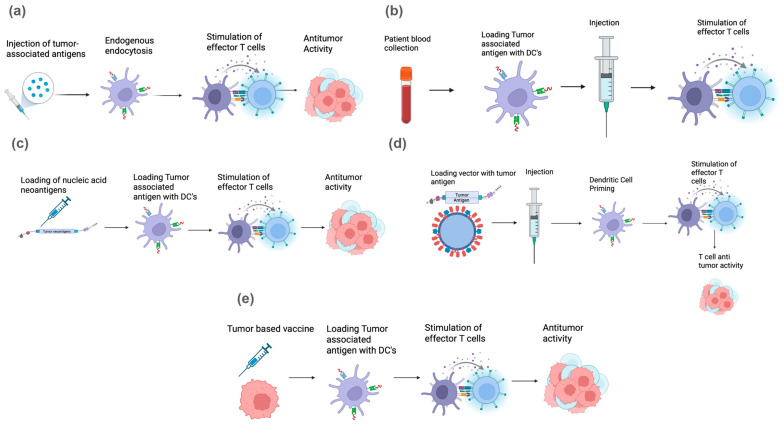
Mechanisms of action underlying key cancer vaccine classes: (**a**) in peptide vaccines, peptides derived from tumor-specific antigens (TAAs) activate effector T-cells; (**b**) in dendritic cell (DC) vaccines, DCs are pulsed with TAAs, which are presented to effector T-cells; (**c**) in nucleic acid vaccines, genetic material encodes TAAs and tumor neoantigens to induce an immune response; (**d**) in vector-based vaccines, immunogenic viral/bacterial vectors are used to deliver the mRNA-encoded TAAs; and (**e**) in whole tumor vaccines, tumor cells are directly used to generate an immune response.

Although there is a myriad of different vaccine categories, they are primarily derived from five main classes in the context of treating malignancy: peptide vaccines, dendritic cell vaccines, whole tumor cell vaccines, viral vector vaccines, and nucleic acid vaccines (Figure 1). These vaccines can target tumor-associated antigens (TAAs), which are host-derived, or neoantigens, which are created from tumor-specific mutations. While each vaccine platform has similarities in its antigen-presenting and downstream immunologic pathways, each class targets tumor cells via distinct mechanisms of action. As such, they each offer advantages and disadvantages in terms of efficacy, safety, and immunogenicity, as we describe below:

### 2.1. Peptide Vaccines

One keystone vaccine strategy in oncology is the peptide vaccine, in which short amino acid sequences are derived from proteins that are either uniquely expressed or overexpressed in tumor cells. One such example of a peptide in the context of GI malignancies is carcinoembryonic antigen (CEA), which becomes emulsified with immunologic adjuvants to enhance uptake and presentation by antigen-presenting cells (APCs) [5] (Figure 1a). The long-peptide vaccines represent a closely related strategy, in which longer amino acid sequences (typically ≥ 20 amino acids) allow presentation by both CD8+ and CD4+ T-cells.

### 2.2. Dendritic Cell Vaccines

Dendritic cell (DC) vaccines leverage DCs’ key role as APCs that prime T cells to coordinate adaptive immunity. Typically, monocyte-derived DCs are harvested from autologous leukapheresis and subsequently loaded in vitro with either a preselected peptide or an mRNA encoding a neoantigen [6]. These modified DCs are then matured with cytokine cocktails before finally being reinfused into the patient (Figure 1b). DC vaccines are of great interest in GI oncology—specifically in treating colorectal carcinoma and pancreatic adenocarcinoma—where endogenous DC activation is often suppressed by the tumor microenvironment (TME) [7].

### 2.3. Whole-Cell Vaccines

Whole-cell vaccines consist of autologous or allogeneic tumor cells that are often irradiated to prevent pathologic replication. These cells are then engineered to secrete immune-stimulatory cytokines, such as granulocyte–macrophage colony-stimulating factor (GM-CSF), which recruits and activates DCs at the site of vaccination (Figure 1e). The prototypical GM-CSF-secreting whole-cell vaccine platform (GVAX) has been tested in both colorectal and pancreatic cancer thus far [8,9]. Whole-cell vaccines of similar formulations are also currently being studied in ongoing trials that are specific to gastric and biliary tract cancers.

### 2.4. Viral Vector Vaccines

Viral vector vaccines utilize viruses that are genetically engineered, so they cannot cause disease but retain the ability to infect host cells. As such, these viruses are modified to encode tumor antigens that are directly presented to host APCs (Figure 1d). This stimulates a strong innate immune response via pattern recognition receptors, including toll-like receptors, thereby enhancing the immune response. One notable example within the field of GI immuno-oncology includes PANVAC, a recombinant vaccinia/fowlpox vaccine encoding CEA [10].

### 2.5. Nucleic Acid Vaccines

Nucleic acid vaccines consist of either a DNA or an mRNA formulation, and they represent a rapidly evolving field of immunology following the advent of the COVID-19 mRNA vaccine [11,12]. DNA vaccines typically deliver plasmid DNA encoding selected antigens and thereby inducing durable cellular immunity. mRNA vaccines, alternatively, are delivered via lipid nanoparticles and translated directly in the cytoplasm to produce the target antigen (Figure 1c). This mechanism eliminates the risk of genomic integration and allows for rapid, customizable manufacturing. Nucleic acid vaccines can also encode multiple neoantigens simultaneously and be co-formulated with immunostimulatory elements, both of which offer flexibility that can be personalized to different patients (a characteristic that is unlike of other vaccine strategies).

## 3. Vaccines in GI Malignancy Types

Of note, while Table 1, Table 2, Table 3 and Table 4 provide an in-depth catalog of trials in which therapeutic cancer vaccines have been implemented to treat GI malignancies, the narrative below will mainly discuss and highlight key landmark trials for each GI cancer subtype. It should be noted that combination regimens composed of both vaccinations and immune checkpoint inhibitors have been rigorously assessed with mechanistic immunological justification prior to implementation in the trials listed below.

### 3.1. Esophageal Cancer

Multiple studies have attempted to implement therapeutic vaccines to treat esophageal malignancies (Table 1). One phase I study evaluated a CHP-MAGE-A4 vaccine in patients (*n* = 22) with primarily metastatic esophageal cancer. The vaccine was found to be safe and able to induce immunogenicity in approximately 25% of patients [13]. Combining multiple immunogenic peptides into a single vaccine has been another strategy explored. For instance, Iinuma et al. administered a 5-peptide vaccine (TTK, URLC10, KOC1, VEGFR1, VEGFR2) along with chemoradiation therapy in patients (*n* = 11) with unresectable ESCC (NCT00632333). The regimen was well tolerated, and several patients experienced prolonged periods of complete response, suggesting a potential role for multi-peptide vaccines given alongside chemoradiation in the setting of locally advanced, unresectable ESCC [14].

**Table 1:** Landmark gastroesophageal vaccines in recent and ongoing trials. Articles accessed on 22 January 2026. An in-depth and comprehensive version of Table 1 is provided in Table S1 in the supplementary section of this manuscript. (AE = adverse event, RFS = relapse-free survival, IFN-γ = interferon gamma, CTL = cytotoxic T-lymphocyte, HER2 = human epidermal growth factor receptor 2, PD-1 = programmed death protein-1).

Compound	Combination Therapy	Primary Outcome	Study Design	Reference
Esophageal Cancer				
CHP-NY-ESO-1	N/A	200 μg induced stronger IgG response (*p* = 0.015) compared to 100 μg	Randomized comparison	[15]
Multiple-peptide vaccine	Radiotherapy, cisplatin, 5-fluorouracil	Grade 3 AEs of neutropenia, anemia, thrombocytopenia observed	Non-comparative observation	[14]
S-588410	N/A	Similar median RFS (84.3 vs. 84.1 months, *p* = 0.8156) in vaccine versus placebo arms	Randomized comparison	[16]
G17DT	Cisplatin, fluorouracil	Over response rate of 30%	Exploratory subgroup analysis	[17]
Neo-DCVac	Neoadjuvant immunotherapy (unspecified)	38.5% with AE without noted grade 3–4 AE	Non-comparative observation	[18]
URLC10-177 VEGFR1-A12-9	N/A	No patient experienced a grade 3–4 AE	Non-comparative observation	[19]
iNeo-Vac-P01	G-CSF	1-, 2-, 3-year RFS rates of 91.3%, 83%, 73.8% (respectively)	Non-comparative observation	[20]
Personalized mRNA tumor vaccine	N/A	AEs	Non-comparative observation (currently in progress)	[21]
Gastric Cancer				
HER-Vaxx (IMU-131)	Standard-of-care chemotherapy	Patients who received 50 µg experienced higher HER2-specific IgG antibody response compared to patients who received 10 µg and 30 µg dosages	Randomized comparison	[22]
HER-Vaxx (IMU-131)	Standard-of-care chemotherapy	Elevation in HER-2 specific IgG and IgG1 antibodies were significantly correlated with tumor reduction (*p* = 0.001 and *p* = 0.016, respectively)	Randomized comparison	[23]
OSTGC-A24	Cyclophosphamide	Positive CTL responses following vaccination were observed in 33% (in 3-week cohort), 88% (in 2-week cohort), and 78% (in 1-week cohort) of patients	Non-randomized observation	[24]
MASCT-I	Camrelizumab	Only AE below grade 2 was reported (e.g., fever, fatigue)	Non-randomized observation	[25]
LY6K-177	N/A	LY6K was expressed in 85% of gastric cancer tissues examined	Non-randomized observation	[26]
p53MVA	N/A	Frequency of PD-1 positive CD8+ T-cells showed an inverse correlation with the peak CD8+ p53 response (*p* = 0.02)	Non-randomized observation	[27]
AD5.F35-GUCY2C PADRE	N/A	Safety/tolerability	Randomized comparison (currently recruiting)	[28]
AST-301	rhuGM-CSF	AEs, IFN-γ assay	Randomized comparison	[29]

S-588410 is a multi-peptide cancer vaccine comprising five HLA-A*24:02-restricted peptides obtained from five cancer-testis antigens. In a phase III study conducted in patients with ESCC who received curative resection after neoadjuvant therapy (*n* = 276), S-588410 showed safety and induced immune responses, but failed to meet the primary endpoint of significantly improved relapse-free survival (84.3 vs. 84.1 weeks, *p* = 0.8156) or overall survival (236.3 weeks and not reached, *p* = 0.6533) when compared against placebo. Overall, 98.5% of vaccinated patients demonstrated CTL responses within 12 weeks, illustrating that while immune responses were achieved, barriers in the TME may have suppressed vaccine-induced CTL antitumor activity. Significantly, among patients with upper thoracic ESCC, grade 3 injection reactions and strong vaccine induced immune responses were associated with prolonged survival. Additionally, S-588410 increased PD-1+ expression in tumor tissues, suggesting that while it did not improve survival as a monotherapy, it may be effective in combination with immune checkpoint inhibitors [16].

Several trials are investigating a novel cancer antigen delivery system, cholesteryl pullulan (CHP), in combination with a cancer-testis antigen such as NY-ESO-1 or MAGE-4. In one phase II trial, a CHP-NY-ESO-1 vaccine was administered to patients (*n* = 54) with surgically resected ESCC at clinical stages II-IV in the adjuvant setting. While no overall improvement in disease-free survival was observed between the vaccine and control groups, patients whose tumors exhibited high NY-ESO-1 expression and elevated polymeric immunoglobulin receptor (PIGR) levels showed greater gene expression changes with vaccination than those with lower expression of these markers [30].

The G17DT vaccine adopts a different strategy by targeting gastrin via stimulating anti-gastrin antibodies. When combined with cisplatin and fluorouracil-based chemotherapy in patients with advanced esophageal and gastric cancer (*n* = 96), the G17DT vaccine improved both overall survival (10.3 months vs. 3.8 months, *p* ≤ 0.0001) and time to tumor progression (5.5 months vs. 2.1 months, *p* = 0.0005) for immune-responders when compared against immune-nonresponders, without any significant increase in treatment-related toxicity. However, it is important to note that the study was a phase II single-arm study with no chemotherapy only control group, suggesting possible confounding results [17].

These trials have demonstrated both safety and immunogenicity, but findings regarding monotherapy efficacy remain limited. Future research aims to focus on combination approaches with immune checkpoint blockade (ICB), as well as on neoantigen discovery to further personalized vaccine development.

### 3.2. Gastric Cancer

Gastric cancer (GC) is the fifth most common malignancy and the fourth leading cause of death globally. Therefore, developing a vaccine remains imperative in treating this specific subtype of GI malignancy. Peptide, nucleic acid, and DC vaccine trials are important avenues of research for GC vaccine development (Table 1). One of the more prominent vaccines against GC is HER-Vaxx (IMU-131), a B-cell epitope peptide vaccine that targets the HER2 oncoprotein. HER-Vaxx induced vaccine-specific immune responses and tumor regression in 11 patients in a phase 1b trial when administered concurrently with chemotherapy in metastatic HER2-positive GC (*n* = 14). In the 11 patients, there was 1 complete response, 5 partial responses, and 4 cases of stable disease. No serious AEs related to vaccination were reported, reflecting a favorable safety profile [22]. In the HERIZON trial, a randomized phase II trial conducted in the metastatic setting, further evidence for the therapeutic potential of HER-Vaxx was demonstrated. The combination of HER-Vaxx and chemotherapy (*n* = 19) led to a 40% improvement in OS, with a median OS of 13.9 months, compared with 8.3 months in the chemotherapy-alone group (*n* = 17). Tumor burden decreased by an average of 30% from baseline in the combination group, compared with a 10% reduction in the chemotherapy-only group [23]. These clinical benefits correlated with robust HER2-specific antibody responses, demonstrating a mechanistic link between humoral immunity and tumor control.

A case report demonstrated the possible benefit of using a personalized neoantigen-loaded monocyte-derived DC vaccine (Neo-MoDC) in combination with PD-1 blockade. Vaccination induced robust T cell responses against neoantigens (NCT03185429), which were further amplified following immune checkpoint inhibition. This approach resulted in a clinical response with complete regression of all tumors for 25 months in a patient [31].

OSTGC-A24 is an HLA-A*24:02-binding vaccine targeting four cancer specific gastric cancer antigens FOXM1, DEPDC1, KIF20A and URLC10, and tumor angiogenesis antigen VEGFR1. In a phase I study, 24 HLA-A*24:02 patients with metastatic disease were enrolled and assigned to three cohorts (*n* = 24) receiving 1 mg of OSTGC-A24 administered every 3 weeks, every 2 weeks, or every week (NCT01227772). The vaccine was well tolerated, and specific CTL induction was achieved with administration every 2 weeks. However, no overall survival or median progression-free survival benefit was demonstrated in the study [24].

Another phase I trial explored the tolerability of combining Mult-Antigen Stimulated Cell Therapy Injection-I (MASCT-I), a multi-antigen peptide vaccine loaded with 15 tumor-associated antigens onto mature DC’s, with the PD-1 antibody camrelizumab in 15 patients with advanced gastric cancer exhibiting PD-L1 expression or microsatellite disease (NCT03393416). There were no grade 3 or 4 adverse reactions, and compared with historical outcomes of camrelizumab therapy, progression-free survival was modestly improved in those who received combination therapy (3.93 months vs. 2 months). Notably, these results are exploratory in nature given the lack of a concurrent control arm and phase I nature of the study [25].

### 3.3. Colorectal Cancer

There are several ongoing vaccine trials for colorectal cancer (CRC). These trials span all five vaccine subtypes mentioned above (Table 2). Notable trials have specifically targeted antigens classically associated with CRC, including CEA and MUC1, whereas more recent trials have employed mutation-directed strategies targeting KRAS or frameshift neoantigens. Early immunogenicity results suggest the potential to reduce recurrence risk in the adjuvant (post-surgical resection) setting, warranting further studies of adjuvant CRC vaccine strategies. Furthermore, because microsatellite-instability CRC has previously been proven to respond well to ICB, it represents an attractive target for ongoing and future combination vaccine strategies.

In a phase II study, the immunogenicity of an RNA-based DC vaccine was compared with that of a peptide-based DC vaccine in the neoadjuvant setting patients with resectable CRC and liver metastases (NCT00228189). Eleven patients received a CEA peptide-pulsed DC vaccine while five received a CEA mRNA-transfected DC vaccine. CEA peptide-specific T cell responses were detected in 8 of 11 patients in the peptide group (73%), whereas no such responses were detected in the RNA group. These findings indicate that DC CEA mRNA transfection was not superior to peptide pulsing for antigen loading of DCs in CRC patients [7].

In a phase I study, 12 patients with metastatic CRC who had progressed on chemotherapy received CEA-pulsed DCs mixed with tetanus toxoid, followed by low-dose IL-2 treatment to increase T cell expansion (NCT00154713). The regimen was well tolerated, with no severe treatment-related adverse events observed. Although T cells showed a significant increase in proliferation against CEA in two patients, the overall immune response was modest [32].

MUC1 mucin is a transmembrane glycoprotein that is overexpressed and hypoglycosylated in colorectal adenocarcinomas and precursor colorectal adenomas compared with MUC1 expressed on normal epithelial cells. A randomized double-blind trial evaluated a MUC1 vaccine versus placebo in individuals with advanced adenomas within 1 year of removal (*n* = 103), assessing whether vaccination could elicit an immune response and reduce colon adenoma formation in patients with high risk for CRC (NCT02134925). At 12 weeks, 25% of vaccine recipients showed a greater than two-fold increase in anti-MUC1 IgG levels compared with placebo, and 22% of vaccine recipients were classified as immune responders at both week 12 and week 55. While the rate of recurrent adenomas was not statistically significant, immune responders experienced a 38% absolute reduction in adenoma recurrence compared with the placebo group [33].

5T4 is a trophoblast glycoprotein with restricted expression in approximately 90% of CRCs. Phase I and II clinical trials evaluated the effectiveness of cyclophosphamide and modified vaccinia Ankara-5T4 to determine whether anti-5T4 immune responses and regulatory T cell depletion were associated with patient survival. Fifty-four patients with metastatic, inoperable CRC were enrolled and randomized to four treatment arms: control, cyclophosphamide only, MVA-5T4 only, or cyclophosphamide plus MVA-5T4. Among those with over a 2-fold increase in anti-5T4 T-cell and antibody responses, cyclophosphamide treatment was associated with longer progression-free survival (5.7 months vs. 2.4 months) and overall survival (20.0 vs. 13.1 months), although these differences did not reach statistical significance (*p* = 0.10 and *p* = 0.09, respectively). Patients who achieved a greater than two-fold increase in anti-5T4 responses had significantly prolonged progression-free (5.6 vs. 2.4 months, *p* < 0.001) and overall survival (20.0 vs. 10.3 months, *p* = 0.008). The addition of cyclophosphamide did not appear to either enhance or diminish the response to the vaccination [34].

**Table 2:** Landmark CRC vaccines in recent and ongoing trials. Articles accessed on 22 January 2026. An in-depth and comprehensive version of Table 2 is provided in Table S2 in the supplementary section of this manuscript. (DC = dendritic cell, CEA = carcinoembryonic antigen, AE = adverse event, PFS = progression-free survival, OS = overall survival, PD-L1 = programmed death-ligand 1, TIL = tumor-infiltrating lymphocyte).

Compound	Combination Therapy	Primary Outcome	Study Design	Reference
CEA-loaded DC	N/A	All patients had T cell responses against control antigen	Exploratory subgroup analysis	[7]
GVAX	Cyclophosphamide, pembrolizumab	Failed to induce radiographic responses, but some biochemical response with CEA decline in 41% of patients	Non-randomized observation	[8]
p53MVA	N/A	CD4+ and CD8+ T cells showing enhanced recognition of p53 peptide, after first vaccination particularly in CD8+ T cells (*p* = 0.03)	Non-randomized observation	[27]
CEA-loaded DC	Low dose interleukin-2	Well tolerated, no severe AEs; two patients had stable disease (17%), 10 patients with disease progression (83%)	Non-randomized observation	[32]
MUC1	N/A	Recurrent adenomas were observed in 66% in placebo group versus 56.3% in MUC1 group (*p* = 0.25)	Randomized comparison	[33]
MVA-5T4	Cyclophosphamide	Patients with greater than 2-fold increase in anti-5T4 responses had prolonged PFS (5.6 vs. 2.4 months, *p* < 0.001) and OS (20.0 vs. 10.3 months, *p* = 0.008)	Randomized comparison	[34]
Alpha-type-1 DC vaccine	N/A	89% of lymphatic cannulations and implantations were successful and well tolerated	Exploratory subgroup analysis	[35]
CV-301	Quadruple Therapy: N-803, M9241, Bintrafusp alfa Triple Therapy: N-803, Bintrafusp alfa	One complete response (5%) in quadruplet therapy arm, 0% in triplet therapy	Non-randomized observation	[36]
GVAX	Guadecitabine	No significant change in CD45RO+ T-cells was found, demonstrating no significant immunologic activity	Exploratory subgroup analysis	[37]
RNA-pulsed DC	N/A	69% of patients had a median relapse at 122 days	Non-randomized observation	[38]
ADC	Best supportive care (BSC)	Median PFS 2.7 months with vaccine versus 2.3 months in control (*p* = 0.628); median OS 6.2 months in vaccine versus 4.7 months in control (*p* = 0.41)	Randomized comparison	[39]
ADC	Avelumab	Study terminated early as primary endpoint was not met (only 11% patients were disease-free at 6 months)	Non-randomized observation	[40]
GRT-C903 GRT-C904	Ipilimumab, nivolumab	Median PFS of 1.9 months, OS of 7.9 months; two patients experienced grade 3/4 AE that were dose-limiting toxicities	Non-randomized observation	[41]
MUC1	N/A	Highly immunogenic in 43.6% of patients, inducing strong anti-MUC1 IgG responses and long-term immune memory	Non-randomized observation	[42]
AlloStim	Cryotherapy	OS greater in AlloStim alone (368 days) compared to AlloStim + cryotherapy (97 days)	Non-randomized observation	[43]
PolyPEPI1018	Atezolizumab	Patients with increased PFS > 12 weeks had increased PD-L1 expression (*p* = 0.007) and increased CD8+ TIL density (*p* = 0.016)	Non-randomized observation	[44]
PolyPEPI1018	N/A	80% of patients had CD8+ T-cell response and patients with multiple doses had PFS 12.5 months and single dose had PFS 4.6 months (*p* = 0.017)	Non-randomized observation	[45]
SPL mKRASvax	Balstilimab, botensilimab	Progression-free survival, objective response rate	Non-randomized observation (currently recruiting)	[46]
NA DC vaccine	Nivolumab	24-mo relapse-free survival, induced immune response against vaccinated NAs	Non-randomized observation (currently recruiting)	[47]

### 3.4. Hepatocellular Carcinoma

Multiple DC vaccine trials have demonstrated both safety and immunogenicity in hepatocellular carcinoma (HCC) (Table 3). However, among the GI malignancies, HCC has arguably demonstrated the most compelling early clinical data regarding personalized vaccine strategies, particularly in combination with anti-PD-1 therapy. One notable small-cohort trial (*n* = 36, NCT04251117) that combined a personalized DNA vaccine (GNOS-PV02) with pembrolizumab reported tumor shrinkage in approximately 30% of patients with advanced HCC, with a favorable safety profile (mainly consisting of injection-site events) [48].

**Table 3:** Landmark HCC vaccines in recent and ongoing trials. Articles accessed on 22 January 2026. An in-depth and comprehensive version of Table 3 is provided in Table S3 in the supplementary section of this manuscript. (ORR = objective response rate, IHC = immunohistochemistry, HLA = human leukocyte antigen, TAA = tumor-associated antigen, DC = dendritic cell, PFS = progression-free survival, AE = adverse event).

Compound	Combination Therapy	Primary Outcomes	Study Design	Reference
GNOS-PV02	Pembrolizumab	ORR of 30.6%, with 8.3% achieving complete response	Non-randomized observation	[48]
GPC3	N/A	Long-term survival at 8 years of 67.1% vs. 38.9% in vaccine arm versus control arm (*p* = 0.038)	Exploratory subgroup analysis	[49]
Hepa-Vac101	Cyclophosphamide	Immune responses against vaccinated HLA class I TAA and vaccinated HLA class II TAA were, respectively, induced in 37% and 53% of patients	Non-randomized observation	[50]
DC vaccine	Transarterial chemoembolization (TACE), cyclophosphamide	Median PFS was 18.6 months in the vaccine infusion arm compared to 10.4 months in the control arm (*p* = 0.016)	Randomized comparison	[51]
HSP70 GPC3	hLAG-3Ig + poly-ICLC	Per IHC analysis, 60% of patients demonstrated infiltration of CD8+ T-cells in tumors with target antigen expression	Randomized comparison	[52]
NA DC	Nivolumab	24-mo relapse-free survival, induced immune response	Non-randomized observation	[47]
CSC DC	N/A	Number of participants with AEs	Randomized comparison	[53]
CRCL + BAG	N/A	Response measured by mRECIST score and experimental biomarker changes	Non-randomized observation	[54]

Glypican-3 (GPC3), a carcinoembryonic antigen, is overexpressed in approximately 80% of hepatocellular carcinomas and has been explored as a target for antigen-specific immunotherapy. The GPC3 peptide vaccine, when used as adjuvant therapy in a phase II trial (*n* = 41), reduced the 1-year recurrence rate by 50% in patients with GPC3-positive tumors. A follow-up study of patients demonstrated longer 5-year and 8-year survival rates in those with GP3C-positive tumors who received the vaccine, attributed to CTLs induced by vaccination [49].

Another peptide vaccine, Hepa-Vac101, incorporates multiple tumor-associated peptides (TUMAPs) presented on HLA molecules of HCC tumor cells and induced tumor-specific T cell responses in approximately two-thirds of patients (*n* = 82, NCT03203005); however, the magnitude of immunogenicity proved to be limited and there was no data on progression-free or overall survival [50]. The ImmunoTACE trial is a randomized phase II trial in patients with intermediate-stage HCC (*n* = 48) that evaluated the efficacy of a DC vaccine in which dendritic cells were pulsed ex vivo with lysates of HepG2 cell lines, in combination with therapeutic transarterial chemoembolization (TACE) and cyclophosphamide preconditioning. Those who received the DC vaccine had improved progression-free survival compared with the controls (18.6 months vs. 10.4 months). The improvement was postulated to result from a combination of TACE-induced antigen release and DC-mediated immune priming, leading to an increased AFP-specific immune response [51].

The YCP02 phase I trial (*n* = 20) employed multi-HLA-binding peptides derived from heat shock protein 70 and glypican-3, combined with a perioperative adjuvant regimen of hLAG-3Ig and Poly-ICLC. The vaccine was well tolerated and demonstrated the potential to convert immunologically “cold” HCC tumors into “hot” tumors through inducing CD8+ T-cell infiltration into tumor tissues. Given its phase I nature, there was no assessment to see if the vaccine improved long-term clinical outcomes [52].

Ongoing and future trials are focused on continuing DC-based approaches and on strategies combining personalized vaccines with ICB (or with procedures such as TACE).

### 3.5. Biliary Tract Cancers

Approaches to treating biliary tract cancers (BTCs), including cholangiocarcinoma and gallbladder adenocarcinoma, primarily consist of early-phase DC and peptide strategies that have demonstrated both safety and feasibility. However, vaccine work in treating BTC remains relatively exploratory, as trial enrollment is limited by both the rarity and heterogeneity of BTC. This is further complicated by the immunosuppressive TME in many BTC cases, which may limit vaccine efficacy. As such, most BTC trials currently remain focused on advancing antigen discovery before progressing to combination trial designs with ICB (Table 4).

**Table 4:** Landmark BTC and PDAC vaccines in recent and ongoing trials. Articles accessed on 22 January 2026. An in-depth and comprehensive version of Table 4 is provided in Table S4 in the supplementary section of this manuscript. (RFS = relapse-free survival, PFS = progression-free survival, OS = overall survival, AE = adverse event, TCR = T-cell receptor, DFS = disease-free survival, IFN-γ = interferon gamma).

Compound	Combination Therapy	Primary Outcomes	Study Design	Reference
Biliary Tract Cancer				
WT1	Gemcitabine	WT1-specific T cells detected in 59% of patients	Non-randomized observation	[55]
OCV-C01	N/A	67% of patients exhibited vaccine-specific T-cell responses to one or more of three antigens	Exploratory subgroup analysis	[56]
HLA-A*24:02 restricted epitope peptides	N/A	Peptide-specific T-cell responses observed in 77% of patients with clinical responses observed in 66% of patients	Non-randomized observation	[57]
Personalized peptide vaccine (PPV)	Cyclophosphamide (CPA)	PPV/CPA arm with significantly higher median PFS (6.1 vs. 2.9 months, *p* = 0.008) and OS (12.1 vs. 5.9 months, *p* = 0.004) compared to PPV alone arm	Randomized comparison	[58]
Elpamotide	Gemcitabine (Gem)	Median survival of 10.1 months, with 1-year survival rate of 44.4%	Exploratory subgroup analysis	[59]
Multiple-peptide vaccine	N/A	Peptide-specific T-cell response observed in 55% of patients	Non-randomized observation	[60]
OBI-833	OBI-821	PFS	Non-randomized observation	[61]
mBTC vax	Durvalumab, tremelimumab	AEs from drug-related toxicities, change in CD8 and CD4 populations	Non-randomized observation	[62]
Pancreatic Adenocarcinoma				
p53MVA	N/A	PD-1 expression limited sustained T-cell expansion (*p* = 0.02)	Non-randomized observation	[27]
GVAX	Cyclophosphamide (Cy), pembrolizumab, stereotactic body radiation	44% of patients underwent resection, with 42% of resected cases showing major pathologic responses	Non-randomized observation	[63]
ELI-002 ELI-002 2P	N/A	Median RFS 16.3 months, which correlated with T cell responses above 12.75-fold above baseline (*p* = 0.0167)	Non-randomized observation	[64]
ELI-002 ELI-002 2P	N/A	At median 19.7 months, patients with mKRAS-specific T cell responses above 9.17-fold above baseline had median radiographic RFS not reached versus 3.02 months (*p* = 0.0002)	Non-randomized observation	[65]
ELI-002 2P	N/A	Phase 1: AEs Phase 2: DFS	Randomized comparison	[66]
Autogene cevumeran	Atezolizumab, mFOLFIRINOX	At 18-month follow-up, T-cell responders had a longer median recurrence-free survival (not reached), compared to non-responders (*p* = 0.003)	Non-randomized observation	[67]
Autogene cevumeran	Atezolizumab, mFOLFIRINOX	At 3.2 year follow up, responders demonstrated longer median recurrence-free survival (not reached) compared with non-responders at 13.4 months (*p* = 0.007)	Non-randomized observation	[68]
Autogene cevumeran	Atezolizumab + mFOLFIRINOX	DFS	Randomized comparison	[69]
SVN-2B	Interferon-β	No significant improvement in PFS (66 days vs. 70 days) compared to control arm (*p* = 0.2617)	Exploratory subgroup analysis	[70]
Mesopher	Mitazalimab	One transient dose-limiting toxicity found; no objective radiographic response observed	Non-randomized observation	[71]
KRAS peptide vaccine + poly-ICLC adjuvant	Nivolumab, ipilimumab	AEs, changes in IFN-γ producing mutant-KRAS-specific CD8+ and CD4+ T-cells	Non-randomized observation	[72]
Algenpantucel-L (NLG0205)	FOLFIRINOX or Gemcitabine/Nab-Paclitaxel	Median OS was 14.9 months in standard group versus 14.3 months in experimental (*p* = 0.98); median PFS 13.4 months in standard and 12.4 months in experimental (*p* = 0.59)	Randomized comparison	[73]
GVAX	Ipilimumab	Combination therapy led to improved survival in 27% of patients along with increased diversification of TCR repertoires	Randomized comparison	[74]
GVAX	Ipilimumab	Median OS 9.38 months in experimental versus 14.7 months in control (*p* = 0.019)	Randomized comparison	[75]
GVAX	Cyclophosphamide (Cy)	GVAX alone had longer DFS than GVAX/Cy combo	Randomized comparison	[76]
GVAX	Nivolumab, stereotactic body radiation (SBRT)	Median OS of 20.4 months, major pathologic response rate of 35%	Non-randomized observation	[77]
GVAX, CRS-207	Cyclophosphamide (Cy), nivolumab	Response rates were not different in treatment arms	Randomized comparison	[78]
Personalized peptide vaccine	Imiquimod, pembrolizumab, sotigalimab	AEs	Non-randomized observation	[79]
KRAS peptide vaccine + poly-ICLC adjuvant	N/A	AEs, changes in IFN-gamma producing mutant-KRAS-specific CD8+ and CD4+ T-cells at 5/13/17 weeks	Non-randomized observation	[80]

Several tumor-associated antigens are being explored as therapeutic targets in BTC. One such target is Wilms’ tumor protein 1 (WT1), which acts as a tumor suppressor and a transcriptional activator of oncogenes [81]. WT1 mutations are present in over 80% of BTC and are associated with poorer prognosis [82]. A phase I trial evaluating a WT1 peptide vaccine in combination with gemcitabine-cisplatin therapy in patients with inoperable advanced biliary tract and pancreatic ductal adenocarcinoma found CTL responses in 59% of patients. In the phase I trial (*n* = 25), median survival time from the first vaccination was approximately 9.5 months among 16 BTC patients, but the lack of a control arm and subsequent phase II data limit the assessment of overall survival or progression-free benefit [55].

Another peptide vaccine strategy is OCV-C01, an HLA-A24:0-restricted, three-peptide cancer vaccine targeting VEGFR1, VEGFR2, and KIF20A. This vaccine targets VEGFR1 and VEGFR2 to inhibit angiogenesis, cancer cell proliferation, and metastasis. In addition, KIF20A, which is highly expressed in cholangiocarcinoma tissues, was included as a tumor-specific antigen. A phase II trial of OCV-C01 was conducted in patients with advanced BTC with unresectable tumors who had failed standard chemotherapy. Six patients were administered the vaccine, four of whom were HLA-A*24:02 positive. These four patients had vaccine-specific T cell responses to one or more of the three antigens. Log-rank analysis demonstrated prolonged overall survival in patients with vaccine-specific T-cell responses compared with non-responders [56].

Another peptide cancer vaccine trial evaluated nine patients with advanced BTC refractory to chemotherapy comprising HLA-A*24:02-restricted epitope peptides which are highly overexpressed in BTC samples: lymphocyte antigen 6 complex locus (LY6K), TTK protein kinase, insulin-like growth factor II mRNA binding protein (IMP-3) and DEP domain-containing 1. This phase I trial revealed that the four-peptide cancer vaccine was well tolerated, with no grade 3 or higher adverse events. Peptide-specific T-cell immune responses were observed in 7 of 9 patients, and stable disease was achieved in 6 of 9. Notably, injection site reactions and CTL induction appeared to be prognostic factors for both overall and progression-free survival, although formal assessment of clinical efficacy was limited given small sample size and lack of a control arm [57].

An open-label randomized phase II study investigated whether low-dose cyclophosphamide could enhance immune and clinical response in patients with BTC receiving personalized peptide vaccinations (PPV). Patients received two to four HLA-restricted peptides selected based on pre-existing peptide-specific IgG responses, and 49 patients were randomized to PPV with or without low-dose cyclophosphamide. PPV combined with low-dose cyclophosphamide improved overall and progression-free survival compared with PPV alone (median OS: 12.1 vs. 5.9 months). Plasma IL-6 increased only in the PPV-alone group, suggesting that cyclophosphamide may enhance anti-tumor immunity by suppressing IL-6 [58].

Regarding ongoing trials, a phase I trial is currently evaluating the safety and immune response of a personalized mutant peptide vaccine with poly-ICLC adjuvant (mBTCvax) in combination with durvalumab and tremelimumab in patients with advanced BTC (NCT06564623).

### 3.6. Pancreatic Adenocarcinoma

Pancreatic ductal adenocarcinoma (PDAC) remains one of the most aggressive malignancies within the field of oncology due to its late-stage diagnosis, with an overall five-year survival of approximately 13% [83]. Although immunotherapy has revolutionized the treatment of pancreatic ductal adenocarcinoma (PDAC), its immunosuppressive TME has historically conferred resistance to multiple immunotherapies [84]. Human PDACs have fewer neoantigens (35 on average) than more immunogenic cancers [85]. Regarding vaccine strategies, numerous studies have been conducted to determine their effectiveness in treating PDAC, with the number of trials published comparable to that in CRC therapy (Table 4). These vaccine strategies have included GVAX (a granulocyte–macrophage colony-stimulating factor-secreting tumor cell vaccine), CRS-207 (a Listeria-based vector), and personalized neoantigen strategies [43,44,45]. One randomized phase 2 trial (*n* = 54, NCT02648282) combining GVAX with CRS-207 failed to show a clear survival benefit over chemotherapy in late-stage disease [63]. However, combinations of vaccines with ICB or with local radiotherapy have produced encouraging immune responses in small-cohort trials. Recent preliminary small-cohort reports highlight neoantigen mRNA vaccine platforms that demonstrate favorable overall survival rates when combined with other forms of multimodality therapy. Ongoing and future studies are expanding on these findings through phase 3 trials with larger cohorts and by designing novel delivery systems, such as lipid nanoparticles for mRNA delivery.

Mutations in KRAS occur in 20–25% of all tumors and are particularly prevalent in very high ratios in PDAC and CRC, with reported rates of 93% and 50%, respectively. The phase 1 AMPLIFY-20 trial evaluated the ELI-002 2 vaccine, a lymph-node-targeted amphiphile vaccine composed of mutant KRAS (mKRAS) peptide antigens targeting the G12D and G12R variants (NCT04853017). The vaccine was given to 25 patients in the adjuvant setting, 20 with PDAC and 5 with CRC, who had detectable minimal residual mutant mKRAS disease after locoregional treatment. ELI-002 2P induced mKRAS-specific T cell responses in 84% of treated patients and was associated with a median tumor biomarker reduction of 76% compared with 10.2% in the control group. Those with robust T cell responses had an 88% reduced risk of relapse compared to those with weaker T cell responses [64]. At one-year follow-up, 67% of patients developed T cell responses against personalized antigens not present in the vaccine. These results suggest that the vaccine led to a broader immune response beyond the initial antigens. Using limited exploratory analysis, the investigators found that patients with significant T cell responses experienced prolonged relapse-free and median overall survival [65]. To further assess whether this vaccine can demonstrate disease-free survival, a randomized phase II trial, AMPLIFY-7P, is underway [66].

Another personalized mRNA neoantigen vaccine is Autogene cevumeran, composed of 20 HLA-restricted neoantigens developed in real time from surgically resected PDAC tumors. In a Phase I study, patients with resectable PDAC received adjuvant atezolizumab, autogene cevumeran, and standard PDAC chemotherapy with modified FOLFIRINOX following surgical resection (*n* = 15, NCT04161755). High-magnitude neoantigen-specific T cell responses were observed in 50% of patients, and at a median follow-up of 18 months, those with vaccine-expanded T cells demonstrated longer median recurrence-free survival than patients without a strong immune response. Additionally, vaccine-expanded T cells persisted for up to 2 years despite subsequent treatment with mFOLFIRNOX [67]. At the 3-year follow-up, responders with vaccine-induced T cells again demonstrated prolonged recurrence-free survival, and 86% of T cell clones in patients persisted at substantial frequencies [68]. While these results showed promising evidence of establishing potentially immunologically active antitumor immune cells, clinical benefit is yet to be determined, with more information on efficacy to be provided in the ongoing IMCODE 003 trial [69].

Survivin is a member of the inhibitor of apoptosis protein (IAP) family and an HLA-A*24-restricted peptide vaccine, HLA-24-SVN-2B, was developed from the survivin splice variant 2B (SVN-2B). This vaccine was evaluated with interferon-β in a phase II clinical trial in patients with unresectable or advanced PDAC. Eighty-three patients were enrolled and randomized to receive the survivin 2B peptide plus interferon-β, SVN-2B alone, or placebo; no progression-free survival was observed across treatment groups. However, among patients who had proceeded in Step 2 of the trial, in which SVN-2B with interferon-β was administered to both groups, those who had received combination therapy of SVN-2B and interferon-B in the initial phase demonstrated improved overall survival [70]. Autopsy analyses from trial participants revealed CTLs had successfully infiltrated tumor lesions; however, high levels of PD-L1 expression on cancer cells were observed, likely limiting antitumor efficacy. These findings suggest that combining a tumor-specific peptide vaccine with an immune checkpoint inhibitor may represent a promising therapeutic strategy [86].

In a phase REACtiVE-2 phase I dose-escalation study, a DC vaccine Mesopher, in combination with the agonistic CD40-specific antibody mitazalimab, was administered to 16 patients with metastatic PDAC following treatment with modified FOLFIRINOX (NCT05650918). Mesopher is an ex vivo-generated DC-based vaccine derived from patients’ monocytes and loaded with allogeneic mesothelioma tumor cell lysate, which contains a broad mix of antigens, including mesothelin, WT1, and survivin, which are expressed in both mesothelioma and PDAC. CD40 activation was used to enhance tumor-specific T cell responses, increase T cell and DC infiltration into tumors, and reduce levels of immunosuppressive M2-like macrophages. Treatment led to vaccine-specific T cell responses, increased intratumoral T cell infiltration, and reduced tumor-associated collagen deposition. However, there were no objective radiological responses, and progression-free and overall survival were not reported as endpoints [71].

## 4. Discussion

The field of therapeutic cancer vaccines in treating GI malignancies has advanced over the past decade, driven largely by advances in tumor genomics, antigen discovery, and combination with other immunotherapy strategies. Despite decades of prior investigation, earlier vaccine efforts were largely restrained by both suboptimal antigen selection and weak immunogenicity.

This contrasts with our contemporary review above, which illustrates a significant shift from single-antigen approaches toward more robust platforms that leverage tumor-specific neoantigens, improved antigen-delivery systems, and synergistic combinations with immune checkpoint blockade and other immunomodulatory therapies.

**Figure 2:**
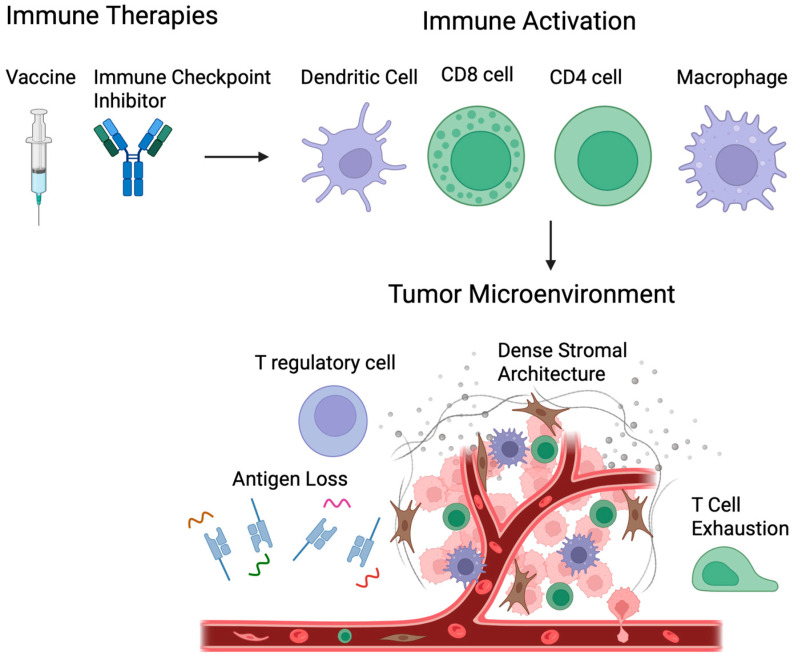
Immune-based therapies within the tumor microenvironment (TME). While vaccines and immunotherapies can activate immune cells and responses, the TME poses formidable barriers such as regulatory T cells, dense stromal architecture, antigen loss and T cell exhaustion, ultimately blunting the immune response.

One central theme that has consistently emerged across the different GI cancer subtypes is the discrepancy between vaccine-induced immunogenicity and clinical significance. Numerous trials have demonstrated an inflammatory response following vaccine administration, including expansion of antigen-specific CD4+ and CD8+ T-cells, increased cytokine production, and (in some cases) enhanced tumor infiltration. However, ORRs and survival improvements remain inconsistent and modest at best in most advanced-disease settings, particularly in early-phase trials. This disconnect highlights the formidable immunosuppressive barriers imposed by the TME, including dense stromal architecture (as seen in the PDAC subtype), regulatory T-cells, and chronic T-cell exhaustion, along with mechanisms intrinsic to tumor evasion such as antigen loss and MHC down-regulation (Figure 2). These factors collectively limit the ability of vaccine-primed T-cells to effectively execute clinically significant anti-tumor activity. In more recent trials, however, immunogenicity has tended to better correlate with overall survival rates; ongoing research should continue to address this discrepancy between appropriate immune response and meaningful clinical benefit.

One such method to address this discrepancy is to consider the clinical context. For instance, there is a growing consensus that minimal residual disease and adjuvant settings represent the most favorable environments for therapeutic vaccination, as seen in various CRC and PDAC trials [67]. Tumor burden is low in these contexts; as a result, immunosuppression is less pronounced, and vaccine-induced immune responses are less likely to be overwhelmed by rapid tumor progression. This mechanism thereby favors earlier immunotherapeutic intervention rather than salvage treatment in end-stage disease.

Furthermore, antigen selection has inherently emerged as a common theme in terms of establishing vaccine effectiveness. Many trials have demonstrated improved immunogenicity and survival with biomarker-driven vaccination strategies, underscoring the importance of continued development of new biomarkers to enable more personalized vaccines, as seen in various HCC and PDAC studies [48,66]. As such, it will remain critical to continue developing new platforms for delivering these vaccines. Of note, across multiple trials, the different vaccine types were overall well tolerated, with few grade 3 adverse events. While injection site reactions were relatively common, they often correlated with improved immune response and longer survival. The prevalence of such adverse effects should continue to be monitored and recorded as research into personalized vaccine development continues.

While many of these studies have shown signals of efficacy, they have predominantly consisted of phase I or phase II data; as such, there remains a need for continued research, especially in phase III and ultimately real-world settings. These studies were also often limited to specific regional medical centers, and it will be crucial to apply these vaccines to a more heterogeneous population. GI cancers are characterized by distinct tumor environments that influence antigen presentation and T-cell infiltration, requiring context-specific approaches. Furthermore, it also remains paramount that ongoing research in treating GI malignancies with cancer vaccines should continue combination trials with ICB, as well as with chemoradiation, in both adjuvant and neoadjuvant settings. Several trials have commented on the combination of ICB with cancer vaccines that can help convert traditionally immunologically “cold” tumors to “hot” tumors via enhancement of T-cell infiltration and modulation of the immunologically suppressive TME. Recent findings reveal that coordination between humoral and cellular immunity can enhance the antitumor effects of ICB, underscoring an important avenue for future investigation [87].

The future roadmap in the development of therapeutic vaccines for GI malignancies will likely be defined by an integrated, comprehensive framework that combines biomarker-guided therapy, advances in artificial intelligence (AI), and next-generation delivery techniques. Biomarkers, ranging from neoantigen load to circulating tumor DNA, will play a central role in identifying patients most likely to benefit from vaccination and subsequent dynamic monitoring of treatment response. Furthermore, AI-driven antigen prediction platforms are concurrently expected to substantially enhance the identification of a variety of neoantigens, thereby enabling the rapid development of more highly personalized vaccines [88]. Finally, both the advances in biomarker- and AI-guided therapy will be complemented by innovations in vaccine delivery platforms. Specifically, lipid nanoparticle-based technology will further optimize delivery efficiency, stability, and targeted uptake by APCs, enhancing both the magnitude and durability of immune responses [89]. Collectively, these developments suggest a future in which the field of GI cancer vaccines becomes robust enough to provide patients with a meaningful form of therapy in addition to traditional, established forms of management.

## 5. Conclusions

Therapeutic cancer vaccines continue to emerge as a potential credible and increasingly sophisticated method of immunotherapy for GI malignancies, driven largely by advances in neoantigen discovery, a multitude of different delivery platforms, and combination strategies with immune checkpoint blockade. Although much of the data is from early and exploratory phases, recent studies have demonstrated that safety, robust immunogenicity, and indicators of clinical efficacy can be achieved, especially when vaccines are deployed under biologically favorable conditions. For instance, the results acquired from these various clinical trials have thus far suggested that successful vaccine strategies in GI oncology will require combination regimens that address tumor microenvironment-mediated immune resistance, as well as strategic deployment in adjuvant or minimal residual disease settings rather than late-stage refractory disease. Continued innovation in this field will also depend heavily on rigorously designed trials that focus on further biomarker and delivery mechanism development. Collectively, these biotechnological advances place cancer vaccines in a landmark period in GI oncology, with the potential to transition from experimental immunologic mechanisms to clinically significant therapies that meaningfully alter the natural progression of GI cancers.

## Data Availability

No new data was created or analyzed in this study.

## Supplementary Materials

The following supporting information can be downloaded at: https://www.mdpi.com/article/10.3390/cancers18091420/s1, Table S1: Landmark gastroesophageal vaccines in recent and ongoing trials (extended); Table S2: Landmark CRC vaccines in recent and ongoing trials (extended); Table S3: Landmark HCC vaccines in recent and ongoing trials (extended); Table S4: Landmark BTC and PDAC vaccines in recent and ongoing trials.
