# Supplementary material for: Therapeutic Cancer Vaccines in Gastrointestinal Malignancies: Advances, Challenges, and Emerging Strategies
Source: Cancers (Basel). 2026 Apr 29;18(9):1420. doi: 10.3390/cancers18091420 (PMC13163029; doi:10.3390/cancers18091420)
Supplement: Supplementary file 1 [file cancers-18-01420-s001.zip › cancers-4229698-supplementary.pdf]

cancers

MDPI

Supplementary Table 1. Landmark gastroesophageal vaccines in recent and ongoing trials (extended). Articles accessed on 22 January 2026. (OS = overall survival, PFS = progression-free survival, AE = adverse event, IFN-γ = interferon gamma, CTL = cytotoxic T-lymphocyte, DFS = disease-free survival, RFS = relapse-free survival, TTP = time to progression, HER2 = human epidermal growth factor receptor 2, PD-L1 = programmed death-ligand 1, PD-1 = programmed death protein-1).

Compound	Mechanism of Action	Combination Therapy	Participants (n)	Phase	Primary Outcome	Other Key Findings	Study Design	Sponsor	Status	NCT Identifier	PMID Number	Reference
Esophageal Cancer												
CHP-NY-ESO-1	NY-ESO-1 cancer-testis antigen loaded with novel antigen delivery system cholesteryl pullulan (CHP)	N/A	25	1	200 µg induced stronger IgG response (p = 0.015) compared to 100 µg	200 µg with similar PFS (p = 0.748) compared to 100 µg; no grade 3-4 AEs observed	Randomized comparison	ImmunoFrontier	Completed	NCT01003808	24093426	[13]
Multiple-peptide vaccine	Peptides included TTK, URLC10, KOC1, VEGFR1, VEGFR2	Radiotherapy, cisplatin, 5-fluorouracil	11	1	Grade 3 toxicities (neutropenia, anemia, thrombocytopenia) observed	All patients developed CTL responses (quantified as IFN-γ); six cases of complete response and five cases of progressive disease observed after the 8th vaccination	Non-comparative observation	Teikyo University	Completed	NCT00632333	24708624	[14]
S-588410	Peptide vaccine comprised of 5 HLA-A*24:02 restricted peptides from five cancer-testis antigens (URLC10, CDCA1, KOC1,	N/A	276	3	Similar median RFS (84.3 vs 84.1 months, p = 0.8156) in vaccine versus placebo arms	Similar OS (p = 0.6533) in vaccine versus placebo arms despite adequate CTL induction; grade 3 injection site reactions reported in 12.9% of patients	Randomized comparison	Takeda Pharmaceutical	Completed	N/A	38990441	[15]

	DEPDC1, MPHOSPH1)										
G17DT	Induction of antigastrin antibody creation	Cisplatin, fluorouracil	103	2	Over response rate of 30%	Median OS and TTP were longer in immune-responders than in immune-nonresponders (p < or = 0.0001 and p = 0.0005, respectively)	Exploratory subgroup analysis	N/A	Completed	N/A	16568451	[18]
Neo-DCVac	Personalized tumor peptide-pulsed dendritic cells	Neoadjuvant immunotherapy (unspecified)	12	1	38.5% with AE without noted grade 3-4 AE	Increased CD3, CD4, and CD8 T-cell populations in all patients; 1-year DFS and OS 88.3% and 100% (respectively); 2-year DFS and OS 66.7% and 91.7% (respectively)	Non-comparative observation	Sichuan University	Completed	NCT05023928	N/A	[24]
URLC10-177 VEGFR1-A12-9	Peptide vaccine containing oncoantigen expressed in esophageal squamous cell carcinoma (eSCC)	N/A	12	1	No patient experienced a grade 3-4 AE	Median OS time of 3.9 months; 4 cases with stable disease and 8 cases with progressive disease following vaccine administration	Non-comparative observation	University of Tokyo	Completed	NCT00753844	24398900	[25]
iNeo-Vac-P01	Personalized cancer vaccine	G-CSF	24	1	1-, 2-, 3-year RFS rates of 91.3%, 83%, 73.8% (respectively)	1-, 2-, 3-year OS rates of 100%, 94.7%, and 81.2%, respectively, T-cell responses in 100% of patients; 4 patients showed 100% response rates	Non-comparative observation	Hangzhou Neoantigen Therapeutics	Completed	NCT05307835	N/A	[26]

Personalized mRNA tumor vaccine	Encodes neoantigen for unresectable or metastatic advanced esophageal cancer	N/A	24	1	AEs	2nd Endpoints: PFS, OS, disease control rate, TTP	Non-comparative observation	Stemirna Therapeutics	In progress	NCT03908671	N/A	[27]
Gastric Cancer												
HER-Vaxx (IMU-131)	B-cell peptide vaccine in HER2-overexpressing gastric adenocarcinoma (induction of HER2-specific antibodies medicating Ab-dependent cell cytotoxicity)	Standard-of-care chemotherapy	11	1	Patients who received 50 µg experienced higher HER2-specific IgG antibody response compared to patients who received 10 µg and 30 µg dosages	Patients who received 50 µg experienced higher PFS compared to patients who received 10 µg and 30 µg dosages	Randomized comparison	Imugene Limited	Completed	NCT02795988	33879458	[19]
HER-Vaxx (IMU-131)	B-cell peptide vaccine in HER2-overexpressing gastric adenocarcinoma (induction of HER2-specific antibodies medicating Ab-dependent cell cytotoxicity)	Standard-of-care chemotherapy	36	2	Elevation in HER-2 specific IgG and IgG1 antibodies were significantly correlated with tumor reduction (p = 0.001 and p = 0.016, respectively)	40% OS benefit with HER-Vaxx with chemotherapy (13.9 months vs. 8.31 months) when compared against chemotherapy alone	Randomized comparison	Imugene Limited	Completed	NCT02795988	39028916	[20]
OSTGC-A24	HLA-A*24:02-binding peptide vaccine cocktail targeting FOXM1, DEPDC1, KIF20A,	Cyclophosphamide	24	1/1b	Positive CTL responses following vaccination were observed in 33% (in 3-week cohort), 88% (in 2-week cohort), and 78% (in 1-week cohort) of patients	Median PFS of 1.7 months and median OS of 5.7 months	Non-randomized observation	National University Hospital, Singapore	Completed	NCT01227772	29587677	[22]

	URLC10 and VEGFR1											
MASCT-I	Autologous non-engineered immune cell therapy composed of multiple-antigen peptides (15 TAAs) loaded mature DCs	Camrelizumab	15	1	Only AE below grade 2 was reported (e.g., fever, fatigue)	Median PFS of 10.23 months (in patients with microsatellite instability) and 2.35 months (in patients expressing PD-L1); longest treatment duration of 18.9 months	Non-randomized observation	HRYZ Biotech	Completed	NCT03393416	N/A	[23]
LY6K-177	Lymphocyte antigen 6 complex locus K (LY6K) expresses a tumor-associated antigen (cancer/testis antigen found in NSCLC and eSCC)	N/A	6	1	LY6K was expressed in 85% of gastric cancer tissues examined	Stable disease in 3 patients, tumor shrinkage in 1 patient following administration; no systemic adverse effects observed	Non-randomized observation	Kinki University	Completed	NCT00845611	23613128	[28]
p53MVA	Genetically engineered version of MVA virus to immunize patients with wild type p53 antigen	N/A	12	1	Frequency of PD-1 positive CD8+ T-cells showed an inverse correlation with the peak CD8+ p53 response (p = 0.02)	No AEs above grade 2 reported following immunization	Non-randomized observation	City of Hope	Completed	NCT01191684	24987057	[29]
AD5.F35-GUCY2C PADRE	Encodes extracellular domain of guanylyl cyclase C, normally restricted to intestinal cells but overexpressed in	N/A	81	2a	Safety/tolerability	2nd Endpoints: T-cell/Ab responses, DFS, OS	Randomized comparison	Thomas Jefferson University	Recruiting	NCT04111172	N/A	[30]

	GI adenocarcinomas											
AST-301	DNA vaccine that encodes the intracellular domain of HER2	rhuGM-CSF	24	2	AEs, IFN-γ assay	2nd Endpoints: DFS, change in memory T-cell populations	Randomized comparison	Aston Sci. Inc.	Recruiting	NCT05771584	N/A	[31]

Supplemental Table 2. Landmark CRC vaccines in recent and ongoing trials (extended). Articles accessed on 22 January 2026. (DC = dendritic cell, CEA = carcinoembryonic antigen, GM-CSF = granulocyte macrophage colony-stimulating factor, AE = adverse event, PD-1 = programmed death protein-1, PFS = progression-free survival, OS = overall survival, TP53 = tumor protein p53, PD-L1 = programmed death-ligand 1, TIL = tumor-infiltrating lymphocyte).

Compound	Mechanism of Action	Combination Therapy	Participants (n)	Phase	Primary Outcome	Other Key Findings	Study Design	Sponsor	Status	NCT Identifier	PMID Number	Reference
CEA-loaded DC	DCs pulsed with CEA-derived HLA-A2 peptide<br><br>DCs electroporated with CEA mRNA	N/A	16	1/2	All patients had T cell responses against control antigen	CEA peptide-specific T cells found in 8 out of 11 patients in peptide group, 0 out of 5 in mRNA group	Exploratory subgroup analysis	Radboud University Medical Center	Completed	NCT00228189	21187495	[7]
GVAX	Whole-cell, GM-CSF immunotherapy inducing T-cell immunity	Cyclophosphamide, pembrolizumab	17	2	Failed to induce radiographic responses, but some biochemical response with CEA decline in 41% of patients	Generally well-tolerated with Grade 3 AEs (hemolytic anemia, corneal transplant rejection) in two patients	Non-randomized observation	Sidney Kimmel (Johns Hopkins)	Completed	NCT02981524	31876399	[8]
p53MVA	Genetically engineered version of	N/A	12	1	CD4+ and CD8+ T cells showing enhanced recognition	The frequency of PD-1 + CD8+ T cells inversely correlated with peak	Non-randomized observation	City of Hope	Completed	NCT01191684	24987057	[29]

	modified vaccinia Ankara (MVA) virus to immunize patients with wild type p53 antigen				of p53 peptide, after first vaccination particularly in CD8+ T cells (p = 0.03)	CD8+ p53 response (p = 0.02)						
CEA-loaded DC	DCs pulsed with CEA	Low dose interleukin-2	12	1	Well-tolerated, no severe AEs; two patients had stable disease (17%), 10 patients with disease progression (83%)	Significant increase in proliferation against CEA by T cells in two patients (22%)	Non-randomized observation	National Institute of Cancer Research, Taiwan	Completed	NCT00154713	27558635	[32]
MUC1	Peptide vaccine containing Mucin-1	N/A	103	2	Recurrent adenomas were observed in 66% in placebo group versus 56.3% in MUC1 group (p = 0.25)	Participants with immune responses in weeks 12 and 55 had a 38% absolute risk reduction in adenoma recurrence compared to placebo (p = 0.08)	Randomized comparison	University of Pittsburgh	Completed	NCT02134925	36892581	[33]
MVA-5T4	Highly attenuated, non-replicating modified vaccinia Ankara virus delivering tumor-associated antigen trophoblast glycoprotein (5T4)	Cyclophosphamide	55	1/2	Patients with greater than 2-fold increase in anti-5T4 responses had prolonged PFS (5.6 vs. 2.4 months, p < 0.001) and OS (20.0 vs. 10.3 months, p = 0.008)	Cyclophosphamide did not enhance the effectiveness of the vaccine	Randomized comparison	Cardiff University	Completed	ISRCTN54669986*	28880972	[34]
Alpha-type-1 DC vaccine	Autologous DCs loaded with killed autologous tumor cells, keyhole limpet hemocyanine (KLH), and PADRE peptides	N/A	9	1	89% of lymphatic cannulations and implantations were successful and well tolerated; average port patency was 7.5 +/- 3.2 weeks	One patient with aggressive carcinomatosis was alive at 90 months without evidence of disease	Exploratory subgroup analysis	Pawel Kalinski	Completed	NCT00558051	27096100	[35]

CV-301	Poxviral vaccine targeting CEA and MUC1	Quadruple Therapy: N-803, M9241, Bintrafusp alfa<br><br>Triple Therapy: N-803, Bintrafusp alfa	32	1/2	One complete response (5%) in quadruplet therapy arm, 0% in triplet therapy	Grade 3 AEs in 8 patients (26.7%) most commonly anemia and GI hemorrhage	Non-randomized observation	National Cancer Institute	Completed	NCT04491955	41520164	[36]
GVAX	Whole-cell, GM-CSF immunotherapy inducing T-cell immunity	Guadecitabine	18	1	No significant change in CD45RO+ T-cells was found, demonstrating no significant immunologic activity	Grade 3-4 AEs were observed to consist of neutropenia, leukopenia, and/or fatigue	Exploratory subgroup analysis	Sidney Kimmel (Johns Hopkins)	Completed	NCT01966289	33531075	[37]
RNA-pulsed DC	CEA mRNA-pulsed patient's DCs are reinfused into the patient's body	N/A	24	2	69% of patients had a median relapse at 122 days	One complete response, two minor responses, three with stable disease and 18 with progressive disease	Non-randomized observation	Duke Cancer Institute	Completed	NCT00003433	23624851	[38]
ADC	Autologous dendritic cells loaded with tumor lysate	Best supportive care (BSC)	52	2	Median PFS 2.7 months with vaccine versus 2.3 months in control (p = 0.628); median OS 6.2 months in vaccine versus 4.7 months in control (p = 0.41)	Among patients with robust immune response, OS increased 7.3 months versus 3.8 months in controls (p = 0.026); study terminated early given futility	Randomized comparison	Fundacion Clinic per a la Recerca Biomedica	Completed	NCT01413295	27428073	[39]
ADC	Autologous dendritic cells	Avelumab	19	1/2	Study terminated early as primary endpoint was not met (only 11% patients were disease-free at 6 months)	Median PFS of 3.1 months and overall survival of 12.2 months; in vitro immune stimulation observed but did not translate into clinical benefit	Non-randomized observation	Grupo Espanol Multidisciplinario del Cancer Digestivo	Completed	NCT03152565	36083313	[40]

GRT-C903 GRT-C904	Chimpanzee adenovirus (ChAd68) primed with self-amplifying mRNA boost	Ipilimumab, nivolumab	39	1/2	Median PFS of 1.9 months, OS of 7.9 months; two patients experienced grade 3/4 AE that were dose-limiting toxicities	T-cell responses skewed towards TP53 neoantigens (included in the vaccine) rather than KRAS, despite KRAS being the most common tumor mutation	Non-randomized observation	Gristone Bio, Inc.	Completed	NCT03953235	38538867	[41]
MUC1	Peptide vaccine (tumor-associated antigen) in setting of advanced colonic adenoma	N/A	39	1/2	Highly immunogenic in 43.6% of patients, inducing strong anti-MUC1 IgG responses and long-term immune memory	No grade 3-4 AE observed in patient with MUC1-specific immune response	Non-randomized observation	Robert Schoen	Completed	NCT00773097	23248097	[42]
AlloStim	Th1 memory cells derived from healthy donor blood using CD3/CD28 microbeads in the setting of third-line metastatic CRC	Cryotherapy	13	2	OS greater in AlloStim alone (368 days) compared to AlloStim + cryotherapy (97 days)	Grade 3 AEs included pancreatitis, fatigue and pneumonia	Non-randomized observation	Mirror Biologics, Inc.	Completed	NCT02380443	N/A	[43]
PolyPEPI1018	Multi-peptide vaccine containing 12 immunogenic epitopes derived from 7 shared tumor antigens	Atezolizumab	18	1b/2a	Patients with increased PFS > 12 weeks had increased PD-L1 expression (p = 0.007) and increased CD8+ TIL density (p = 0.016)	Disease control rate of 61%; 81.3% showed robust vaccine CD4/CD8 T-cell response	Non-randomized observation	Treos Bio Limited	Completed	NCT05243862	N/A	[44]
PolyPEPI1018	Multi-peptide vaccine containing 12 immunogenic epitopes derived from 7 shared tumor antigens	N/A	11	2b	80% of patients had CD8+ T-cell response and patients with multiple doses had PFS 12.5 months and single dose had	Well tolerated vaccine with no serious AEs reported	Non-randomized observation	Tres Bio Zrt	Completed	NCT03391232	35472243	[45]

					PFS 4.6 months (p = 0.017)							
SPL mKRASvax	Peptide vaccine targeting KRAS	Balstilimab, botensilimab	54	1	Progression-free survival, objective response rate	Secondary Endpoint: Disease control rate	Non-randomized observation	Sidney Kimmel (Johns Hopkins)	Recruiting	NCT06411691	N/A	[46]
NA DC vaccine	Neoantigen dendritic cell vaccine	Nivolumab	60	2	24-mo relapse free survival, induced immune response against vaccinated NAs	Secondary Endpoints: AEs, OS	Non-randomized observation	Bristol-Myers Squibb	Recruiting	NCT04912765	N/A	[47]

* This trial is registered in the UK's clinical study registry (ISRCTN).

Supplemental Table 3. Landmark HCC vaccines in recent and ongoing trials (extended). Articles accessed on 22 January 2026. (ORR = objective response rate, IHC = immunohistochemistry, CTL = cytotoxic T-lymphocyte, HLA = human leukocyte antigen, TAA = tumor-associated antigen, DC = dendritic cell, PFS = progression-free survival, AE = adverse event, OS = overall survival).

Compound	Mechanism of Action	Combination Therapy	Participants (n)	Phase	Outcomes	Other Key Findings	Study Design	Sponsor	Status	NCT Identifier	PMID Number	Reference
GNOS-PV02	DNA plasmid personalized therapeutic cancer vaccine	Pembrolizumab	36	1/2	ORR of 30.6%, with 8.3% achieving complete response	Neoantigen-specific T-cell response in 86.4%; no dose-limited toxicities or grade 3 adverse events noted	Non-randomized observation	Geneos Therapeutics	Completed	NCT04251117	38584166	[48]
GPC3	Glypican-3 peptide vaccine	N/A	68	2	Long-term survival at 8 years of 67.1% vs. 38.9% in vaccine arm versus control arm (p = 0.038)	Patients positive for GPC3 IHC staining are more likely to have induced CTLs, with 60% survival beyond 5 years	Exploratory subgroup analysis	Onco Therapy Science, Inc	Completed	UMIN000002614*	32449239	[49]
Hepa-Vac101	Combination of IMA970A (5 HLA-A*24 and 7 HLA-A*02 as well as 4 HLA-DR	Cyclophosphamide	82	1/2	Immune responses against vaccinated HLA class I TAA and vaccinated HLA class II TAA were	Grade 3-4 AEs in 2 patients: non-serious amylase increase, lipase increase, influenza-like illness	Non-randomized observation	National Cancer Institute, Naples	Completed	NCT03203005	35421231	[50]

	restricted peptides) as well as CV8102 (RNA-based immunostimulator)				respectively induced in 37% and 53% of patients						
DC vaccine	Monocyte-derived autologous DCs pulsed with a cell lysate made from HepG2 cells (a hepatoblastoma cell line known to express several putative HCC tumor antigens)	Transarterial chemoembolization (TACE), cyclophosphamide	48	2	Median PFS was 18.6 months in the vaccine infusion arm compared to 10.4 months in the control arm (p = 0.016)	The addition of DC infusions did not significantly increase the incidence of severity of AEs	Randomized comparison	NIHR Birmingham Liver Biomedical Research Centre.	Completed	09/160/24**	40499144	[51]
HSP70 GPC3	Peptide vaccine containing heat shock protein 70 and glypican-3	hLAG-3Ig + poly-ICLC	20	1	Per IHC analysis, 60% of patients demonstrated infiltration of CD8+ T-cells in tumors with target antigen expression	Grade 1 AEs in 95% of patients	Randomized comparison	CYTLIMIC	Completed	jRCTs061180058***	36929310	[52]
NA DC	Neoantigen dendritic cell vaccine	Nivolumab	60	2	24-mo relapse free survival, induced immune response	2nd Endpoints: AEs, OS	Non-randomized observation	National Cancer Centre, Singapore	Recruiting	NCT04912765	N/A	[47]
CSC DC	Cancer stem cell-loaded dendritic cell vaccine	N/A	40	1/2	# of participants with AEs	2nd Endpoints: Vaccine immune responses	Randomized comparison	Fuda Cancer Hospital, Guangzhou	Active	NCT02089919	N/A	[53]
CRCL + BAG	Chaperone-rich cell lysate (calreticulin, HSP70, HSP90, gp96 neoantigens) derived from patient's own tumor biopsy + bioengineered	N/A	15	2	Response measured by mRECIST score and experimental biomarker changes	2nd Endpoints: Immune monitoring via longitudinal CT scans/biopsies	Non-randomized observation	Mirror Biologies, Inc.	Active	NCT02409524	N/A	[54]

	allograft (derived from healthy donor Th1 memory cells)

* This trial is registered in the Japanese University Hospital Medical Information Network (UMIN). ** This trial is registered in the British clinical study registry (NIHR). *** This trial is in Japan's Registry for Clinical Trials (jRCT).

Supplementary Table 4. Landmark BTC and PDAC vaccines in recent and ongoing trials (extended). Articles accessed on 22 January 2026. (GM-CSF = granulocyte macrophage colony-stimulating factor, RFS = relapse-free survival, PFS = progression-free survival, OS = overall survival, AE = adverse event, TCR = T-cell receptor, DFS = disease-free survival, TSA = tumor-specific antigen, TAA = tumor-associated antigen, ORR = objective response rate, IFN-γ = interferon gamma).

Compound	Mechanism of Action	Combination Therapy	Participants (n)	Phase	Primary Outcome	Other Key Findings	Study Design	Sponsor	Status	NCT Identifier	PMID Number	Reference
						**Biliary Tract Cancer**						
WT1	Wilms tumor 1 peptide vaccine	Gemcitabine	25	1	WT1-specific T cells detected in 59% of patients	2-month disease control rate of 50% with median survival time of 288 days	Non-randomized observation	Jikei University School of Medicine	Completed	UMIN000004081*	21150717	[57]
OCV-C01	HLA-A*24:02-restricted three-peptide cancer vaccine targeting VEGFR1, VEGFR2 (targets both tumor angiogenesis) as well as KIF20A (oncoantigen)	N/A	6	2	67% of patients exhibited vaccine-specific T-cell responses to one or more of three antigens	T-cell response was significantly correlated with overall survival (p = 0.0177)	Exploratory subgroup analysis	Cancer Precision Medicine, Inc.	Completed	UMIN000012778*	33788741	[58]
HLA-A*2402 restricted	Four-peptide vaccine containing lymphocyte	N/A	9	1	Peptide-specific T-cell responses observed in 77%	Median PFS and OS of 156 and 380 days,	Non-randomized observation	Nakayama Cancer	Completed	UMIN000003207*	23479678	[59]

epitope peptides	antigen 6 complex locus K-177, TTK protein kinase-567, insulin-like growth factor-II mRNA-binding protein 3-508, DEP domain containing 1				of patients with clinical responses observed in 66% of patients	respectively; no grade 3 or 4 adverse events following four-peptide vaccination		Research Institute				
Personalized peptide vaccine (PPV)	Maximum of four HLA class I-matched peptides are selected from candidate antigen peptides	Cyclophosphamide (CPA)	49	2	PPV/CPA arm with significantly higher median PFS (6.1 vs. 2.9 months, p = 0.008) and OS (12.1 vs. 5.9 months, p = 0.004) compared to PPV alone arm	Increase in IL-6 observed only in PPV/CPA arm	Randomized comparison	Taiho Pharmaceutical Co., Ltd.	Completed	UMIN000006249*	28188670	[60]
Elpamotide	HLA-A*24:02-restricted epitope peptide that induces CTLs to specifically recognize VEGFR-2/KDR169	Gemcitabine (Gem)	55	2	Median survival of 10.1 months, with 1-year survival rate of 44.4%	Injection site reactions were observed in 64.8%, in whom median survival was 14.8 months compared to 5.7 months in those with no injection site reaction (p = 0.002)	Exploratory subgroup analysis	OncoTherapy Science	Completed	UMIN000002500*	25502982	[61]
Multiple-peptide vaccine	Targeted antigens included CDCA1, KIF20A, cadherin 3 (derived from cancer-testis antigens) within patients with HLA type A*2402	N/A	9	1	Peptide-specific T-cell response observed in 55% of patients	Median PFS and OS of 3.4 and 9.7 months, respectively; no grade 3-4 AEs	Non-randomized observation	University of Tokyo	Completed	UMIN000003229*	24606884	[62]

OBI-833	Carbohydrate-conjugate vaccine designed to induce complement-dependent cytotoxicity (mediated by IgM and IgG) against tumor cells expressing the Globo H antigen	OBI-821	30	2	PFS	Secondary Endpoints: OS, tumor response, safety profile	Non-randomized observation	Chang Gung Memorial Hospital	Not yet recruiting	NCT06490198	N/A	[63]
mBTC vax	Peptide vaccine with poly-ICLC (dsRNA complex) that activates toll-like receptor 3 to enhance dendritic and NK cell activity	Durvalumab, tremelimumab	25	1	AEs from drug-related toxicities, change in CD8 and CD4 populations	Secondary Endpoint: PFS and OS	Non-randomized observation	AstraZeneca	Recruiting	NCT06564623	N/A	[64]
Pancreatic Adenocarcinoma												
p53MVA	Genetically engineered version of MVA virus to immunize patients with wild type p53 antigen	N/A	12	1	PD-1 expression limited sustained T-cell expansion (p = 0.02)	Safe, well-tolerated and immunogenic with robust CD8 T-cell responses against p53	Non-randomized observation	City of Hope	Completed	NCT01191684	24987057	[29]
GVAX	Vaccine introduces irradiated pancreatic tumor cells that have been genetically	Cyclophosphamide (Cy), pembrolizumab, stereotactic body radiation	58	2	44% of patients underwent resection, with 42% of resected cases showing major pathologic responses	Combination therapy was feasible and immunologically active in locally advanced	Non-randomized observation	Sidney Kimmel (Johns Hopkins)	Completed	NCT02648282	38324679	[68]

	modified to produce GM-CSF					pancreatic cancer						
ELI-002 ELI-002 2P	A peptide-based cancer vaccine that consists of amphiphile KRAS peptides combined with CpG adjuvant	N/A	25	1	Median RFS 16.3 months, which correlated with T cell responses above 12.75-fold above baseline (p = 0.0167)	84% of patients demonstrated mKRAS-specific T-cell responses; no dose-limiting toxicities were observed	Non-randomized observation	Elicio Therapeutics	Completed	NCT04853017	38195752	[69]
ELI-002 ELI-002 2P	A peptide-based cancer vaccine that consists of amphiphile KRAS peptides combined with CpG adjuvant	N/A	25	1	At median 19.7 months, patients with mKRAS-specific T cell responses above 9.17-fold above baseline had median radiographic RFS not reached versus 3.02 months (p = 0.0002)	71% of patients induced both CD4 and CD8 cells, and antigen spreading present in 67% of patients	Non-randomized observation	Elicio Therapeutics	Completed	NCT04853017	40790272	[70]
Autogene cevumeran	An mRNA neoantigen vaccine generated in real time from surgically resected PDAC tumors	Atezolizumab, mFOLFIRINOX	29	1	At 18-month follow-up, T-cell responders had a longer median recurrence free survival (not reached), compared to non-responders (p = 0.003)	Vaccine-induced clonal expansion was detected in 100% of responders and in 12.5% of non-responders	Non-randomized observation	Memorial Sloan Kettering Cancer Center	Completed	NCT04161755	37165196	[72]
Autogene cevumeran	An mRNA neoantigen vaccine generated in real time from	Atezolizumab, mFOLFIRINOX	29	1	At 3.2 year follow up, responders demonstrated longer median	In responders, the vaccine induced CD8 T-cell clones with	Non-randomized observation	Memorial Sloan Kettering Cancer Center	Completed	NCT04161755	39972124	[73]

	surgically resected PDAC tumors				recurrence-free survival (not reached) compared with non-responders at 13.4 months (p = 0.007)	average lifespan 7.7 years						
SVN-2B	A peptide vaccine developed from survivin splice variant 2B (SVN-2B)	Interferon-β	83	2	No significant improvement in PFS (66 days vs. 70 days) compared to control arm (p = 0.2617)	Subgroup analysis demonstrated that longer SVN-2B with IFN-β treatment may lead to improved survival	Exploratory subgroup analysis	Sapporo Medical University	Completed	UMIN000012146*	31218770	[75]
Mesopher	Autologous DC vaccine	Mitazalimab	16	1	One transient dose-limiting toxicity found; no objective radiographic response observed	50% of patients showed stable disease after three administrations	Non-randomized observation	Erasmus MC Cancer Institute	Completed	NCT05650918	41315287	[77]
Algenpantucel-L (NLG0205)	Introduction of irradiated allogenic pancreatic cancer cells transfected to express murine α-1,3-galactosyltransferase, with subsequent rapid activation of antibody-dependent cell-mediated cytotoxicity	FOLFIRINOX or Gemcitabine/Nab-Paclitaxel	303	3	Median OS was 14.9 months in standard group versus 14.3 months in experimental (p = 0.98); median PFS 13.4 months in standard and 12.4 months in experimental (p = 0.59)	Grade 3 or 4 AEs 75% in standard and 81% in experimental (p > 0.05)	Randomized comparison	NewLink Genetics	Completed	NCT01836432	33630475	[79]

GVAX	Vaccine introduces irradiated pancreatic tumor cells that have been genetically modified to produce GM-CSF	Ipilimumab	25	1	Combination therapy led to improved survival in 27% of patients along with increased diversification of TCR repertoires	Patients with more than 100 clones expanded after treatment survived nearly 3 times longer than patients with fewer clones expanded ($p < 0.01$)	Randomized comparison	Sidney Kimmel (Johns Hopkins)	Completed	NCT00836407	29997287	[80]
GVAX	Vaccine introduces irradiated pancreatic tumor cells that have been genetically modified to produce GM-CSF	Ipilimumab	82	2	Median OS 9.38 months in experimental versus 14.7 months in control ($p = 0.019$)	Combination therapy promoted T-cell differentiation and increased M1 macrophages in tumor; study discontinued due to futility after analysis	Randomized comparison	Sidney Kimmel (Johns Hopkins)	Completed	NCT01896869	32591464	[81]
GVAX	Vaccine introduces irradiated pancreatic tumor cells that have been genetically modified to produce GM-CSF	Cyclophosphamide (Cy)	87	2	GVAX alone had longer DFS than GVAX/Cy combo	Tertiary lymphoid aggregates developed in 85% patients after vaccination	Randomized comparison	Sidney Kimmel (Johns Hopkins)	Completed	NCT00727441	33277370	[82]
GVAX	Vaccine introduces irradiated pancreatic tumor cells that have been genetically modified to produce GM-CSF	Nivolumab, stereotactic body radiation (SBRT)	31	2	Median OS of 20.4 months, major pathologic response rate of 35%	There was no difference in mean CD8 T cell density in study patients versus historical control	Non-randomized observation	Sidney Kimmel (Johns Hopkins)	Completed	NCT03161379	40407726	[83]

GVAX, CRS-207	GVAX = irradiated pancreatic tumor cells that have been genetically modified to produce GM-CSF<br><br>CRS-207 = live, attenuated *Listeria monocytogenes* expressing mesothelin	Cyclophosphamide (Cy), nivolumab	57	2	Response rates were not different in treatment arms	Grade 3+ AEs were seen in 39 (68%) patients, with 33 of those attributed to CRS-207	Randomized comparison	Sidney Kimmel (Johns Hopkins)	Completed	NCT03190265	41524570	[84]
Personalized peptide vaccine	Induces TSA/TAA-specific effector T-cell immune responses	Imiquimod, pembrolizumab, sotigalimab	150	1	AEs	OS, PFS, RFS, response rate, changes in neoantigen-specific T-cell response	Non-randomized observation	MD Anderson Cancer Center	Enrolling by invitation	NCT02600949	N/A	[85]
KRAS peptide vaccine + poly-ICLC adjuvant	A peptide-based cancer vaccine against mutant KRAS	N/A	25	1	AEs, changes in IFN-gamma producing mutant-KRAS-specific CD8+ and CD4+ T-cells at 5/13/17 weeks	Changes in T-cell quality	Non-randomized observation	Sidney Kimmel (Johns Hopkins)	Recruiting	NCT05013216	N/A	[86]
KRAS peptide vaccine + poly-ICLC adjuvant	A peptide-based cancer vaccine against mutant KRAS	Nivolumab, ipilimumab	30	1	AEs, changes in IFN-γ producing mutant-KRAS-specific CD8+ and CD4+ T-cells	OS, DFS, PFS, ORR	Non-randomized observation	Sidney Kimmel (Johns Hopkins)	Active	NCT04117087	N/A	[78]
ELI-002 2P	A peptide-based cancer vaccine that consists of amphiphile KRAS	N/A	158	1/2	Phase 1: AEs<br>Phase 2: DFS	Phase 1: Reduction in ctDNA, CA 19-9, CEA	Randomized comparison	Elicio Therapeutics	Active	NCT05726864	N/A	[71]

	peptides combined with CpG adjuvant					Phase 2: OS						
Autogene cevumeran	An mRNA neoantigen vaccine generated in real time from surgically resected PDAC tumors	Atezolizumab + mFOLFIRINOX	260	2	DFS	OS, AEs	Randomized comparison	Genentech, Inc.	Recruiting	NCT05968326	N/A	[74]

* These trials are registered in the Japanese University Hospital Medical Information Network (UMIN).
